# Tanshinone IIA Attenuates Insulin Like Growth Factor 1 -Induced Cell Proliferation in PC12 Cells through the PI3K/Akt and MEK/ERK Pathways

**DOI:** 10.3390/ijms19092719

**Published:** 2018-09-12

**Authors:** Haitao Wang, Xiaoying Su, Jiankang Fang, Xingan Xin, Xia Zhao, Uma Gaur, Qiang Wen, Jiangping Xu, Peter J. Little, Wenhua Zheng

**Affiliations:** 1 Faculty of Health Science, University of Macau, Taipa, Macau 999078, China; wht821@smu.edu.cn (H.W.); yb57646@umac.mo (J.F.); yb77638@connect.umac.mo (X.X.); yb77625@umac.mo (X.Z.); gaur.uma2906@gmail.com (U.G.); 2 School of Pharmaceutical Sciences, Sothern Medical University, Guangzhou 510515, China; jpx@smu.edu.cn; 3 School of Pharmaceutical Sciences, Sun Yat-Sen University, Guangzhou 510006, China; sxy811@gmail.com (X.S.); wenqiang998@gmail.com (Q.W.); 4 School of Pharmacy, Pharmacy Australia Centre of Excellence, The University of Queensland, Woolloongabba, Queensland 4102, Australia; p.little@uq.edu.au

**Keywords:** Tanshinone IIA, antiproliferation, IGF-1R, Akt, ERK

## Abstract

The insulin like growth factor 1 (IGF-1) and its receptor (IGF-1R) facilitate tumor proliferation and progression. Tanshinone IIA (TSN) is an active diterpene quinone isolated from the roots of the herbal plant *Salvia miltiorrhiza*. TSN inhibits the proliferation of various types of cancer cells but its role in the IGF-1R-induced proliferation of pheochromocytoma (PC12) cells and the potential mechanisms are largely unknown. This study aims to investigate the anti-proliferative effect of TSN in PC12 cells and its role on IGF-1R signaling transduction. PC12 cells were treated with IGF-1 with or without TSN, methyl thiazolytetrazolium (MTT) assay, and cell counting kit-8 and flow cytometry were used to evaluate the proliferation of PC12 cells. The role of TSN on the apoptosis of PC12 cells were detected by flow cytometry as well. The effects of TSN and IGF-1 on the phosphorylation of IGF-1R, protein kinase B (Akt), extracellular-signal related kinase 1/2 (ERK1/2) and other downstream targets were analyzed by Western blotting analysis. Our results showed that IGF-1 promoted the growth of PC12 cells in a dose-dependent manner and increased the phosphorylation of IGF-1R, whereas TSN attenuated the effect of IGF-1. Interestingly, TSN did not induce cell apoptosis in PC12 cells. Moreover, TSN attenuated the phosphorylation of Akt and ERK1/2 induced by IGF-1, and the phosphorylation of glycogen synthase kinase-3β, forkhead box O3a (FOXO3a) and c-Raf were also inhibited by TSN. Furthermore, TSN inhibited cell growth induced by IGF-1 and blocked the activation of IGF-1R in SH-SY5Y cells. Taken together, TSN has an inhibitory effect on the proliferation of PC12 cells via down-regulation of the phosphorylated IGF-1R and its downstream signaling.

## 1. Introduction

**Figure 1:**
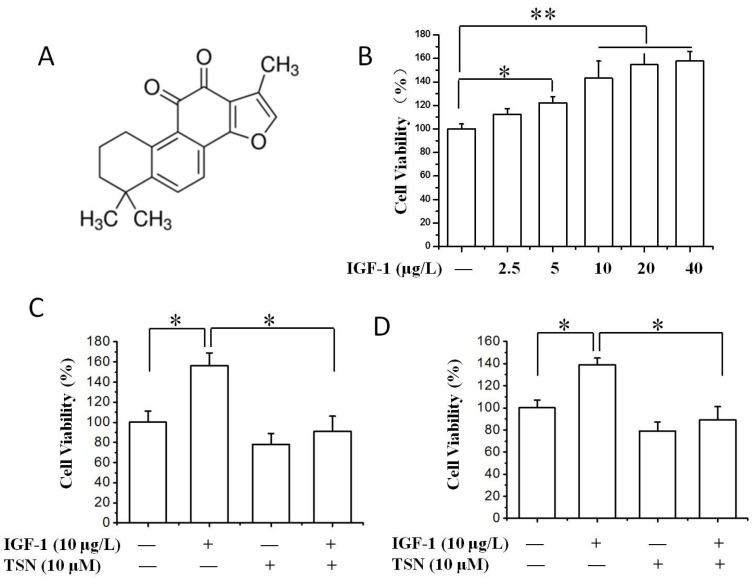
Tanshinone IIA (TSN) attenuated IGF-1-induced cell proliferation in PC12 cells. (**A**) Chemical structure of TSN; (**B**) The effect of IGF-1 on PC12 cell proliferation. The cells were treated with various concentrations of IGF-1 (0–40 μg/L) in a serum-free medium for 24 h; cell proliferation was evaluated by methyl thiazolytetrazolium (MTT) assay; (**C**) Effect of TSN on IGF-1-induced cell proliferation in PC12 cells. Cells were treated with TSN (10 μM) for 1 h, and then were treated with IGF-1 in a serum-free medium for 24 h. Cell proliferation was determined by MTT assay; (**D**) Cells were treated with TSN and IGF-1 in a serum-free medium for 24 h. Cell proliferation was determined by CCK-8 assay. Each graph represents data from triplicates in separate experiments. Values were expressed as mean ± SEM. * *p* < 0.05, ** *p* < 0.01.

Tanshinone IIA (TSN) is one of the active ingredients extracted from *Salvia miltiorrhiza Bunge* (*S. miltiorrhiza*), which is also called Danshen in China. *S. miltiorrhiza* has been shown to increase coronary blood flow, inhibit platelet aggregation and scavenge the free radicals in ischemic diseases [1], and that is why it has been used for the prevention and treatment of cardio-cerebral vascular diseases in Asian countries [2,3,4]. Additionally, it has also displayed a therapeutic potential in the treatment of Alzheimer’s disease via multiple mechanisms, including anti-aggregation of amyloid peptides [5], anti-apoptosis [6], acetylcholinesterase inhibition [7], and anti-inflammation [5,8]. Many active ingredients have been identified from *S. miltiorrhiza*, such as tanshinone I, TSN and salvianolic acids [9]. TSN is an active diterpene quinone isolated from the root of *S. miltiorrhiza*. The chemical structure of TSN is shown in Figure 1A. Interestingly, TSN is also shown to dilate blood vessels, attenuate myocardial ischemia injury and is thereby viewed as a promising compound for the prevention and treatment of cardiovascular diseases [10]. It should be noted that TSN also has some toxicity concerns. Studies showed that 4-days treatment with TSN caused a dose-dependent abnormality of heart (pericardial edema and spinal curvature) in zebra fish embryos. The mortality of TSN at 24 μM for four days was 51.6% [11]. Another study also suggested that TSN at high concentration (≥320 μM) induced significant cytotoxicity in both HEK293 cells and rat vascular smooth muscle cells [12]. This data proposes the potential cytotoxic effect of TSN, and we should pay more attention to these side effects during drug research and development. Effective toxicological screening models, such as the zebra fish, make an ideal animal model to evaluate the acute toxicity and developmental toxicity of TSN.

In addition to the pharmacological effects mentioned above, recent studies have highlighted the anti-cancer potential of *S. miltiorrhiza* [13]. Among multiple active ingredients in *S. miltiorrhiza*, TSN has attracted increasing attention from the research community due to its potent inhibitory effect on the growth of tumor cells in a variety of cancer cell lines, including lung cancer [14], colon cancer [15], breast and prostate cancer cells [16,17]. Mechanistic studies indicate that TSN inhibits cell growth by decreasing the activity of cyclin-dependent kinases (CDKs), leading to cell cycle arrest [18,19]. The mammalian target of rapamycin (mTOR) /ribosomal protein S6 kinase (p70S6K) pathway and p38/Jun-amino-terminal kinase (JNK) pathway are proposed to be highly associated with the anti-cancerous activities of TSN in various cancer cell lines [18,20]. A recent study showed that TSN suppresses tumor growth by increasing the levels of pro-apoptotic proteins and decreasing the expression of anti-apoptotic molecules [19]. In addition, TSN may also decrease the activity of matrix metalloproteinase 2 and play an anti-angiogenic effect [21]. The mitochondrial dysfunction and the subsequent dynamic alterations are also involved in the inhibitory effect of TSN on tumor angiogenesis [19]. Taken together, increasing evidence indicates that TSN is a potential anti-cancer agent and the underlying mechanisms are just beginning to be uncovered.

Insulin-like growth factor l (IGF-l) is a polypeptide trophic factor, which plays important roles in the regulation of cell survival, proliferation and differentiation of various cells [22,23]. The biological functions of IGF-l are mainly mediated by IGF-l receptors (IGF-1R). The binding of IGF-1 to its receptors causes the phosphorylation/activation of IGF-1R, and subsequently activates downstream signal transduction [24]. The phosphatidylinositol-3-kinase/protein kinase B (PI3K/Akt) and mitogen-activated protein kinase (MAPK) signaling pathways are two major pathways that mediate the effects of IGF-1/IGF-1R [25,26,27]. Recent studies have suggested that the level of circulating IGF-1 is higher in patients with pheochromocytoma [28], glioma [29], breast cancer or prostate cancer [30]. IGF-1R is also seen highly activated in tumor cells [31]. IGF-1 is a strong mitogen, which stimulates IGF-1R signaling and thus plays important roles in the occurrence and growth of several cancers [30]. Similar to its roles in physiological conditions, IGF-1R promotes mitogenesis and tumorigenesis by activating a variety of signaling pathways, including the PI3K/Akt and the MAPK pathways. IGF-1R has become an attractive therapeutic target due to its oncogenic role caused by the aberrant signaling [32]. One of the effective ways to block signaling from IGF-1R in cancer cells is to identify the small molecules which can inhibit the tyrosine phosphorylation of its β subunit. TSN is an active molecule, and it has been proven to inhibit the growth of several cancer cells [14,18,19,20]. However, the inhibitory potential of TSN in IGF-1 and IGF-1R-mediated tumorigenesis has not yet been studied in detail.

In the present report, we have studied the effect of TSN on the proliferation of rat pheochromocytoma (PC12) cells induced by IGF-1 and its underlying mechanisms. The role of TSN on the IGF-1R activation was also verified in SH-SY5Y cells. The results showed that IGF-1 promoted the proliferation of PC12 cells in a dose-dependent manner, induced the phosphorylation of IGF-1R and activated its downstream signaling pathways. Whereas, TSN showed a significant inhibitory effect on the IGF-1 induced proliferation of PC12 cells. TSN attenuated the phosphorylation of IGF-1R in both PC12 and SH-SY5Y cells. The inhibitory effect of TSN is mediated by attenuating the phosphorylation of IGF-1R along with its downstream signaling pathway.

## 2. Results

### 2.1. TSN Suppressed Cell Growth Induced by IGF-1 in PC12 Cells

IGF-1 is a growth factor which can stimulate the proliferation of cancer cells dramatically [30], and IGF-1R is highly expressed in PC12 cells [33]. In the present study, PC12 cells were firstly treated with different concentrations of IGF-1 ranging from 0 to 40 μg/L, and the stimulatory effect of IGF-1 on the growth of PC12 cells was measured by thiazolyl blue tetrazolium blue (MTT) assay and cell counting kit-8 (CCK-8). We found that 0–40 μg/L IGF-1 dramatically enhanced the cell viability of PC12 cells and increased the value of optical density in MTT assay (Figure 1B). We further explored the role of TSN on cell viability in the presence of IGF-1. As shown in Figure 1C, treatment with TSN for 24 h significantly suppressed the value of optical density in PC12 cells. To confirm the inhibitory effect of TSN on cell viability, CCK-8 was used and the results were consistent with MTT assay (Figure 1D). Taken together, the data indicated that TSN exerted an anti-proliferative effect on PC12 cells induced by IGF-1.

### 2.2. TSN had No Effect on the Apoptosis of PC12 Cells

**Figure 2:**
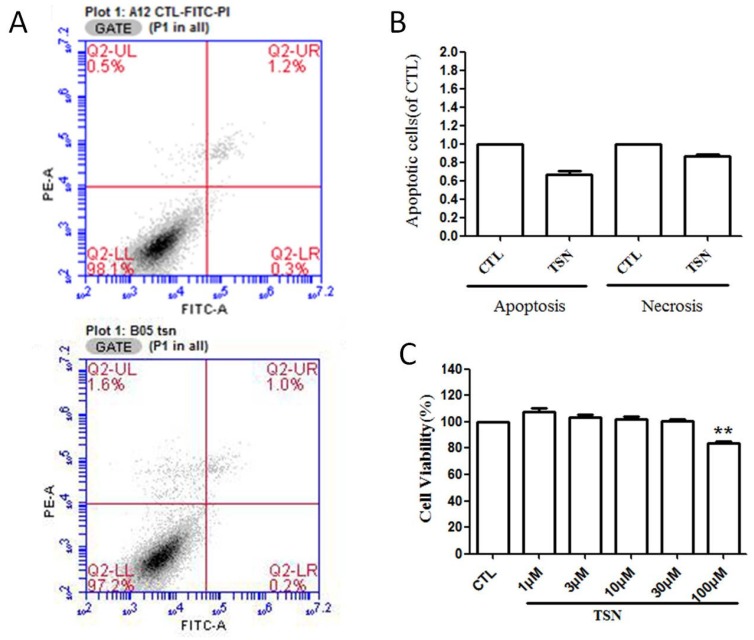
TSN had no effect on cell apoptosis in PC12 cells. Cells were pretreated with or without TSN (20 μM) for 24 h. The apoptosis of PC12 cells was determined by flow cytometry. (**A**) Photographs of representative cultures measured by Flow cytometry; (**B**) Quantification of apoptotic cells; (**C**) PC12 cells were treated with various concentrations (1–100 μM) of TSN for 24 h and cell viability was measured using MTT assay. The values were expressed as mean ± SEM. ** *p* < 0.01, compared with control. CTL, Contorl.

Having known that TSN attenuated the cell viability of PC12 cells in the presence of IGF-1, we then wanted to know whether TSN would affect the apoptosis of PC12 cells. Flow cytometry was used to evaluate the early and late apoptosis that occurred in PC12 cells after treatment with TSN (10 μM) for 24 h. We found that there was no significant difference between the control group and the TSN group (*p* > 0.05) (Figure 2A,B). We also treated cells with various concentrations of TSN (1–100 μM) and cell viability was measured by MTT assay. Our results indicated that TSN produced no toxicity at the concentration less than 100 μM (Figure 2C). Taken together, Cytotoxicity assays in PC12 cells showed that TSN did not induce necrosis/apoptosis of PC12 cells at the doses used for the present study.

### 2.3. TSN Inhibited IGF-1-Induced Tyrosine Phosphorylation of IGF-1R in PC12 Cells

**Figure 3:**
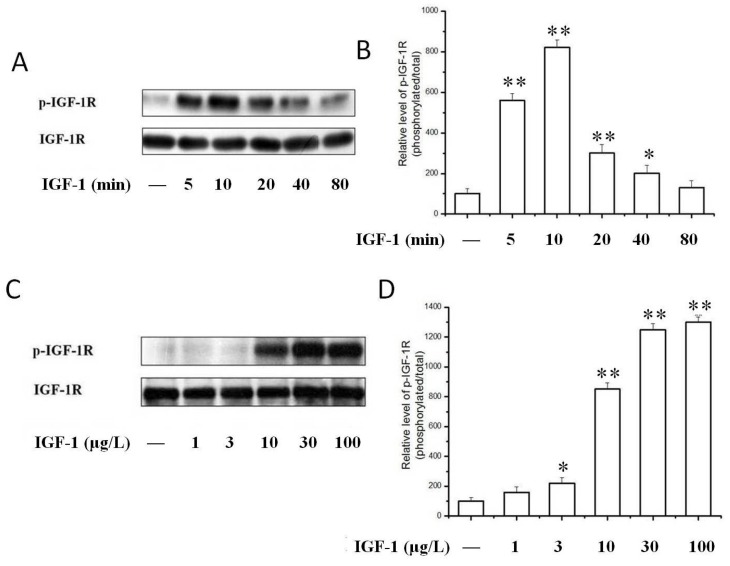
IGF-1 time- and dose-dependently activated IGF-1R. (**A**) PC12 Cells were treated with 10 μg/L IGF-1 for various times and the phosphorylation of IGF-1R was determined by Western blotting; (**B**) The ratio of p-IGF-1R/IGF-1 in PC12 cells after treatment with 10 μg/L IGF-1 for various time; (**C**) Cells were treated with various concentration of IGF-1 for 10 min and the phosphorylation of IGF-1R was determined by Western blot; (**D**) The ratio of p-IGF-1R/IGF-1 in PC12 cells after treatment with various concentrations of IGF-1 for 10 min. Results are shown as the mean ± SEM and blots represent experiments performed in triplicates. * *p* < 0.05, ** *p* < 0.01 versus control.

**Figure 4:**
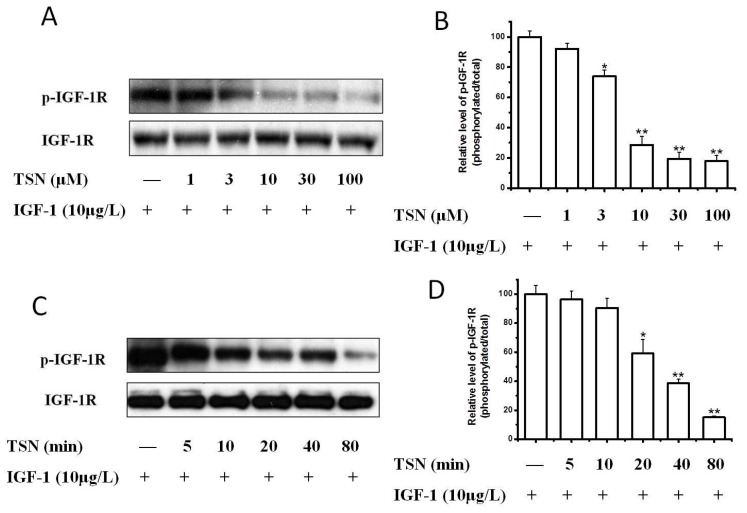
TSN attenuated IGF-1R activation induced by IGF-1 in PC12 cells. (**A**) PC12 cells were treated with various concentrations of TSN and 10 μg/L IGF-1. The levels of p-IGF-1R were determined by Western blotting; (**B**) The ratio of p-IGF-1R/IGF-1R in PC12 cells after treatment with various concentration of TSN and 10 μg/L IGF-1; (**C**) PC12 cells were treated with 20 μM TSN and 10 μg/L IGF-1 at various time points. The levels of p-IGF-1R were determined by Western blotting; (**D**) Relative levels of p-IGF-1/IGF-1R in PC12 cells treated with 20 μM TSN and 10 μg/L IGF-1 at various time points were determined by densitometry of the blots and densitometric analysis of the immunoblot was expressed as a percentage of control. The results are displayed as the mean ± SEM and represent three independent experiments, * *p* < 0.05, ** *p* < 0.01 versus control.

Having demonstrated that IGF-1 prompted the proliferation of PC12 cells, we next investigated the signaling pathways possibly responsible for this effect. We investigated IGF-1-stimulated tyrosine phosphorylation of the IGF-1R, which is the initial and essential step of IGF-1 signaling. Compared to the serum-free control, IGF-1 concentration-dependently stimulated the tyrosine phosphorylation of IGF-1R in PC12 cells (Figure 3). IGF-1 (10 μg/L) stimulated the tyrosine phosphorylation of IGF-1R at various time points ranging from 5 to 80 min (Figure 3A,B). The phosphorylation of IGF-1R reached a peak value within 10 min and declined afterwards. We thus selected this time point for subsequent studies. The phosphorylation of IGF-1R decreased after 20 min, but the phosphorylation level of IGF-1R was still higher than the basal level for about 40 to 80 min. Consistently the effect of IGF-1 on the phosphorylation of IGF-1R was found to be concentration-dependent (Figure 3C,D). The tyrosine phosphorylation of IGF-1R in PC12 cells was observed at a concentration of 3 μg/L IGF-1 and increased as the concentration of IGF-1 increased to a maximum of 100 μg/L. We then explored whether TSN had an inhibitory effect on the activation of IGF-1R in PC12 cells. As shown in Figure 4A, when cells were co-treated with TSN (1–100 μM) and IGF-1 in serum-free medium, TSN inhibited phosphorylation of IGF-1R at Tyr1135/Tyr1136 in a dose-dependent manner in PC12 cells (Figure 4A,B), which was consistent with the inhibition on cell proliferation. Furthermore, TSN at a dose of 20 μM suppressed the phosphorylation of IGF-1R in a time-dependent manner (Figure 4C,D). Therefore, this data suggested that IGF-1 induced a rapid phosphorylation of IGF-1R in PC12 cells, whereas TSN significantly attenuated the tyrosine phosphorylation of IGF-1R in a time- and concentration-dependent manner.

### 2.4. TSN Attenuated the Activation of Akt and MAPK Induced by IGF-1

**Figure 5:**
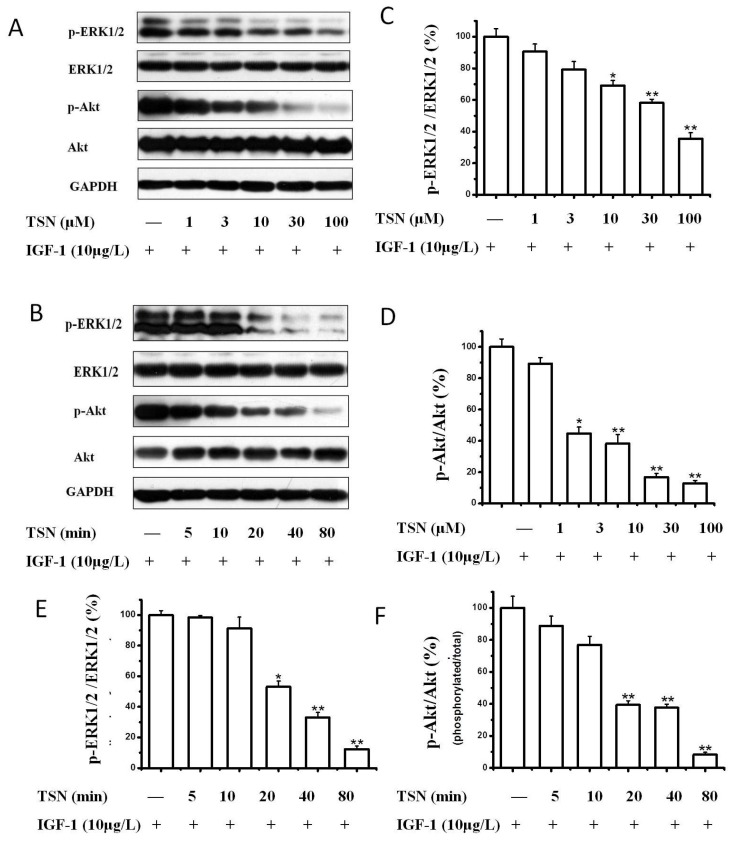
TSN attenuated the activation of Akt and ERK1/2 in PC12 cells in a dose- and time-dependent manner. (**A**) Cells were pre-treated with various concentrations of TSN, and then incubated with 10 μg/L IGF-1 for 10 min. The expressions of p-Akt and p-ERK1/2 were determined by Western blotting; (**B**) Cells were pretreated with 20 μM TSN at various time points and then incubated with IGF-1 for 10 min. The expressions of p-Akt and p-ERK1/2 were determined by Western blotting; (**C**–**F**) Relative levels of p-Akt versus total Akt and p-ERK1/2 versus ERK1/2 in each sample were determined by the densitometry of the blots. Densitometric analysis of the blots was expressed as a percentage of control. The results are shown as the mean ± SEM and represent three independent experiments, * *p* < 0.05, ** *p* < 0.01 versus control.

We further sought to find out whether PI3K/Akt and MAPK pathways were involved in the anti-proliferative action of TSN in IGF-1 stimulated PC12 cells, as these two are the main signaling pathways mediating the biological functions of IGF-1R. PC12 cells were pre-treated with various concentrations of TSN (1–100 μM) for 60 min, and then incubated with IGF-1 (10 μg/L) for 10 min. The extent of phosphorylation of Akt and extracellular signal–regulated kinases 1/2 (ERK1/2) was determined by Western blotting. The results showed that TSN attenuated the activation of Akt in PC12 cells in a dose-dependent manner, which was consistent with tyrosine phosphorylation of IGF-1R induced by IGF-1 (Figure 5A,D). Similar results were observed for the phosphorylation of ERK1/2 (Figure 5A,C). We also tested the time-course action of TSN. As shown in Figure 5B, PC12 cells were treated with 20 μM TSN for various time points (0–80 min) and then stimulated with 10 μg/L of IGF-1 for 10 min. Both Akt and ERK phosphorylation was appreciably blocked (Figure 5E,F), indicating that TSN not only inhibits the phosphorylation of IGF-1R, but also inhibits the IGF-1R-mediated signaling pathways.

### 2.5. TSN Inhibited IGF-1R Mediated Akt and Mitogen-Activated Protein Kinase Kinase (MEK) Signaling Transduction

**Figure 6:**
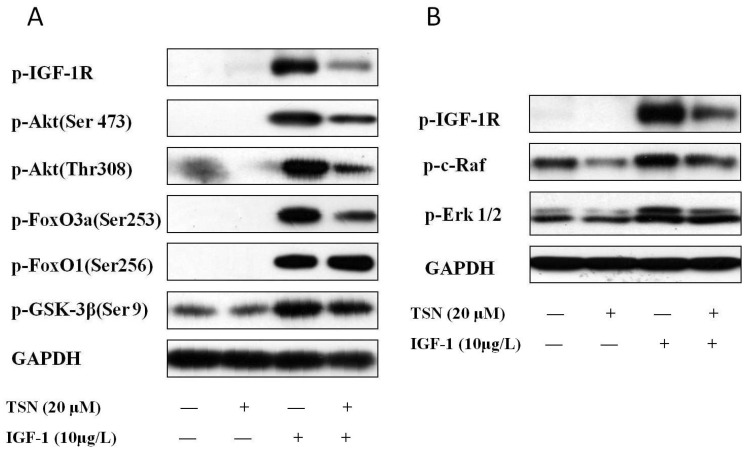
TSN inhibits IGF-1R mediated Akt and MEK signaling transduction. (**A**) PC12 cells were treated with 20 μM TSN and 10 μg/L IGF-1. The levels of p-IGF-1R, p-Akt, p-GSK-3β, p-FoxO3a and p-FoxO1 were determined by Western blotting; (**B**) PC12 cells were treated with 20 μM TSN and 10 μg/L IGF-1. The levels of p-c-Raf and p-ERK1/2 were determined by Western blotting.

Having known that TSN not only inhibited the phosphorylation of IGF-1R, but also inhibited the activation of Akt and ERK1/2, we next focused on the downstream signaling of Akt and ERK1/2. The downstream target of Akt, including glycogen synthase kinase-3 beta (GSK-3β), forkhead box O1 (FoxO1) and forkhead box O3a (FoxO3a) were detected, as these are highly related to cellular proliferation [34]. Upstream kinase c-Raf was also detected through Western blot, as it is involved in the IGF-1 signaling for the promotion of cell proliferation [35]. As shown in Figure 6A, the treatment of PC12 cells with IGF-1 dramatically increased the phosphorylation of Akt at both Ser473 and Thr308, which lead to the full activation of Akt [36]. Moreover, the downstream targets of Akt, including GSK-3β, FoxO3a and FoxO1 were also phosphorylated. Although TSN blocked the activity of IGF-1 and attenuated the levels of phosphorylated Akt, GSK-3β and FoxO3a, it had no effect on the phosphorylation of FoxO1 (Figure 6A), indicating that GSK-3β and FoxO3a mediated the main anti-proliferative effect of TSN. Furthermore, we found that IGF-1 also had a significant effect on the phosphorylation of c-Raf (Figure 6B), which is an upstream kinase of ERK1/2. Taken together, the data suggested that TSN has dual effects on PI3K/Akt/GSK-3β/FoxO3a signaling as well as c-Raf/MEK/ERK signaling.

### 2.6. TSN Inhibited IGF-1-Induced Cell Growth and Tyrosine Phosphorylation of IGF-1R in SH-SY5Y Cells

**Figure 7:**
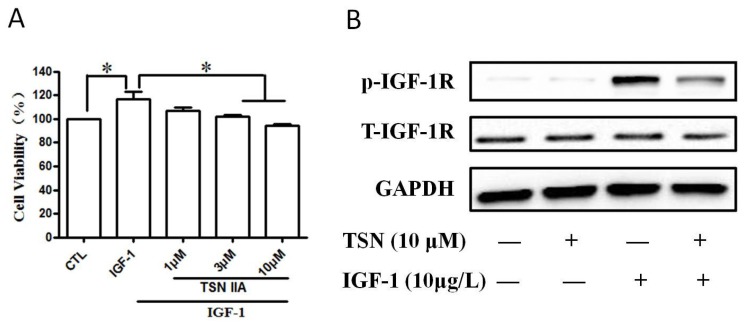
TSN inhibited cell growth induced by IGF-1 and attenuated the phosphorylation of IGF-1R in SH-SY5Y cells. (**A**) Cells were treated with TSN (10 μM) for 1 h, and were then treated with IGF-1 in a serum-free medium for 24 h. Cell proliferation was determined by MTT assay; (**B**) SH-SY5Y cells were treated with various concentrations of TSN for 1 h, and followed by treatment with 10 μg/L IGF-1. The levels of p-IGF-1R and IGF-1R was determined by Western blot. The data is expressed as mean ± SEM, *n* = 3. * *p* < 0.05, compared to control.

Our above study is performed with rat cell line. To assume more evidence, we also verified the inhibitory role of TSN on cell growth in a human neuroblastoma cell line SH-SY5Y cell. SH-SY5Y cells were pre-treated with various concentrations (1–10 μM) of TSN for 60 min and then stimulated with IGF-1 for 24 min. As shown in Figure 7, we found that IGF-1 enhanced the cell viability of SH-SY5Y cells and increased the value of optical density in MTT assay (Figure 7A). Treatment with TSN for 24 h significantly suppressed the value of optical density in SH-SY5Y cells (Figure 7A). We further explored the role of TSN on the phosphorylation of IGF-1R in SH-SY5Y cells in the presence of IGF-1. We found that TSN decreased the phosphorylation of IGF-1R in SH-SY5Y cells (Figure 7B), which was consistent with the results obtained in PC12 cells. This data indicates that TSN is effective in blocking the activation of IGF-1R induced by IGF-1 in the SH-SY5Y cells.

## 3. Discussion

**Figure 8:**
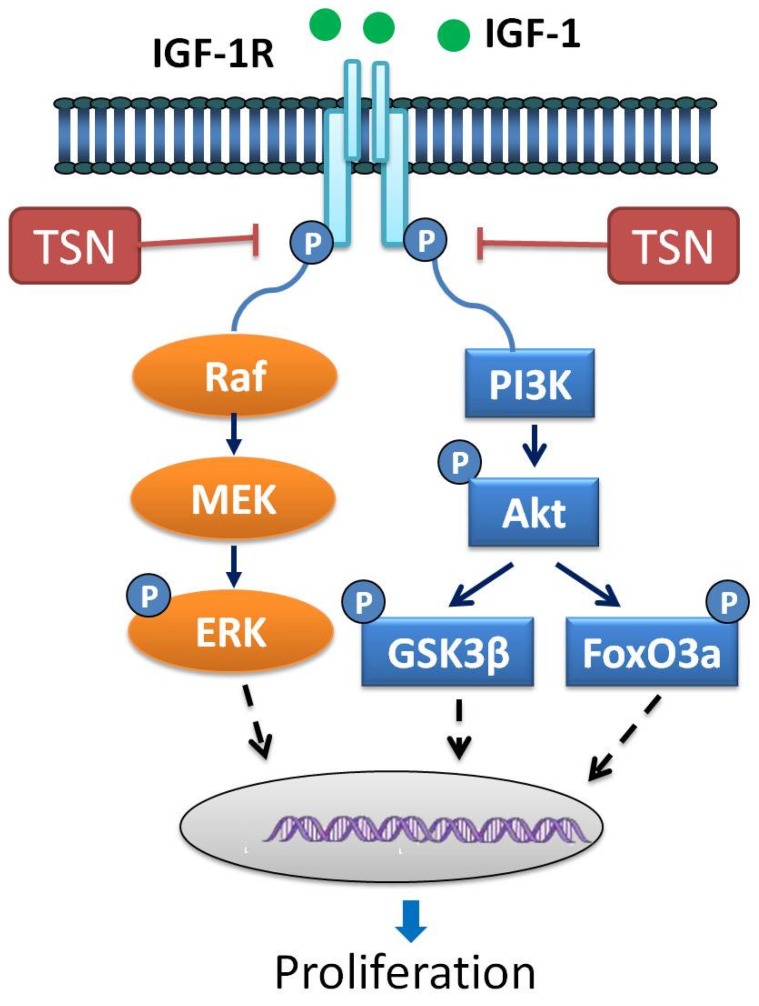
Schematic diagram of signaling mechanisms involved in the effects of TSN on cellular proliferation induced by IGF-1. IGF-1R is highly expressed in various cancer cells. When exposed to IGF-1, IGF-1R is auto-phosphorylated and activated. The activated IGF-1R subsequently regulates PI3K/Akt and MEK/MAPK signaling pathways, leading to cellular proliferation. TSN blocks the tyrosine phosphorylation of IGF-1R stimulated by IGF-1. As a consequence, TSN exerts its anti-proliferative effects by inhibiting IGF-1R and thus PI3K/Akt and MEK/MAPK signaling pathways.

In the present study we tried to explore the anti-proliferative effect of TSN PC12 and SH-SY5Y cells stimulated with IGF-1. We found that: (1) IGF-1 stimulated the proliferation of PC12 cells in a dose-dependent manner, while TSN blocked the role of IGF-1; (2) TSN had no effect on the apoptosis of PC12 cells below the dose of 30 μM; (3) treatment with TSN attenuated the activation of IGF-1R induced by IGF-1; (4) IGF-1 activated PI3K/Akt and ERK1/2 pathways in PC12 cells, while pretreatment with TSN attenuated the activation of IGF-1R downstream signaling. Therefore, it is plausible to propose that the anti-proliferative action of TSN is mainly mediated through inactivating IGF-1R as well as PI3K/Akt and ERK1/2 pathways. A schematic representation of the present work is shown in Figure 8. As far as we know, this is the first work that shows the anti-proliferative effect of TSN in cancer cells treated with IGF-1. Our study provides a novel mechanism illustrating the anti-cancer role of TSN.

Emerging evidence suggests that IGF-1 and IGF-1R signaling play a pivotal role in the oncogenic transformation, growth and survival of a variety of cancers, including prostate cancer, breast cancer, colon cancer, and myeloma [23,30,31]. The pro-proliferative effect of IGF-1 is mainly mediated by the phosphorylation of IGF-1R tyrosine kinases. Phosphorylation of IGF-1R ultimately phosphorylates MAPK and Akt, and the signal cascade is finally transmitted to the nucleus, initiating gene expression to promote cell proliferation. The binding of IGF-1 to its receptor IGF-1R resulted in the increased activation of tyrosine kinases, and this IGF-1R activation initiated various downstream cascades such as the PI3K/Akt and Ras/ERK1/2 signaling pathways [34]. The activated Akt subsequently phosphorylates GSK-3β directly, and both Akt and ERK1/2 are direct upstream kinases of FoxO3a [37]. The role of GSK-3β in cancer remains complex and controversial since GSK-3β may act as a tumor-promoter as well as tumor-suppressor [38]. In our study, we found that IGF-1 stimulated the phosphorylation of GSK-3β in PC12 cells, indicating that the inhibition of GSK-3β plays a positive role in promoting the development of cancer, at least in IGF-1R-mediated cancer. This result was consistent with the finding that the inhibition of GSK-3β induced invasion in breast cancer [39]. The possible mechanism behind this may be that the phosphorylation of GSK-3β causes much more stabilization of beta-catenin, which promotes the expression of cyclin-B1 and survivin, and thereby promotes the proliferation and survival of cancer cells [38]. On the other hand, we found that IGF-1 also inactivated FoxO3a in our research. Phosphorylated FoxO3a is mainly localized in the cytoplasm which reduces its ability to contact and regulate its target genes, such as the Bcl-2-interacting mediator of cell death and the p53 upregulated modulator of apoptosis [40]. Thus, suppressing IGF-1R might be an effective therapeutic strategy to attenuate the proliferation of cancer cells, especially for the cancer cells which have higher expressions of IGF-1R.

The identification of molecular targets involved in the process of carcinogenesis represent a rational approach for the therapeutic intervention of cancer. In recent years, compounds targeting the IGF-1R have emerged as a matter of great interest to researchers [41]. In the present study, TSN obviously reduced the IGF-1R tyrosine phosphorylation. Moreover, it also inhibited the activation of Akt and ERK. Interestingly enough we found that GSK-3β and FoxO3a were also important participants in the anti-proliferative effect of TSN. Previous studies have also indicated that TSN inhibited the growth of cancer cells through CDKs/cyclin, p38/JNK and mTOR/p70S6K [18,19,20]. Looking at all this evidence, we concluded that TSN is a multi-target drug for cancer therapy. We also proposed a new mechanism for the inhibitory effect of TSN on cancer cell proliferation. Our research will also enhance the understanding of the therapeutic application of TSN for cancer patients. The present work emphasizes the inhibitory effect of TSN on IGF-1R and the downstream signal transduction. Interestingly, IGF-1R activation is capable of mediating the activation of mTOR/p70S6K both in vitro and in vivo and endogenous mTOR/p70S6K is tyrosine-phosphorylated in response to IGF-1 stimulation [42]. IGF-1R is also linked to the activation of Akt/mTOR signaling and activation of JNK in cancer cells [43]. Hence, in PC12 and SH-SY5Y cells, the relationship between IGF-1R inhibition and other signaling pathways related to cell growth deserves to be investigated in future research. Additionally, under the condition that TSN inhibited the activation of IGF-1R, a rescue experiment should be performed to determine if over-expression of IGF-1R could rescue the TSN-induced inhibition of proliferation. An appropriate positive control should also be taken into consideration in these experiments. This will be our extended work in future studies.

In our study, we found that TSN blocked the pro-proliferative effect role of IGF-1 in PC12 and SH-SY5Y cells, and we verified that TSN attenuated the activation of IGF-1R and the subsequent signaling molecules. However, how TSN affects the activation of IGF-1R is still unknown. TSN may act directly on the phosphorylation site of IGF-1R and block the binding of IGF-1 to IGF-1R. Alternatively, TSN may also act on protein phosphatase and thus promote the dephosphorylation of kinases, including IGF-1R. The interaction of TSN and IGF-1R is deserved to be studied extensively in the future. On the other hand, the data presented here is mainly obtained from cell lines; the role of TSN on IGF-1R and the involved cell signaling warrant an investigation in tumor animal models treated with TSN in the future.

Taken together, the data presented here indicated that TSN exhibited a potent ability to block the IGF-1-stimulated activation of IGF-1R, its downstream signaling like the Akt/GSK-3β/FoxO3a and ERK1/2 pathways and cell growth. These results suggest that TSN may be a potential drug in the treatment of cancer cells highly expressing IGF-1R such as pheochromocytoma and glioblastoma.

## 4. Materials and Methods

### 4.1. Materials

TSN was obtained from National Institutes for Food and Drug Control (Beijing, China), IGF-1, dimethyl sulfoxide (DMSO), poly-l-lysine and bovine serum albumin was the product of Sigma (St. Louis, MO, USA). PC12 cells were given by Dr. Gordon Guroff (National Institute of Child Health and Human Development, National Institutes of Health, Bethesda, MD, USA), and SH-SY5Y cells were from the Cell Resources Bank of the Laboratory Animal Center, Sun Yat-sen University. Fetal bovine serum (FBS), antibiotics, trypsin and Dulbecco’s Modified Eagle’s Medium (DMEM) were purchased from Invitrogen (Carlsbad, CA, USA), MTT was purchased from Sigma (St. Louis, MO, USA), Cell Counting Kit-8 was from DOJINDO Laboratories (Shanghai, China). Anti-GAPDH, anti-IGF-1R, anti-phospho-IGF-1R (Tyr1135/Tyr1136), anti-phospho-FoxO3a (Ser253), anti-phospho-FoxO1 (Ser256) were from Signalway Antibody LLC (Baltimore, MD, USA), anti-phospho-GSK-3β(Ser9) was from BioLabs (Ipswich, NE, USA), anti-Phospho-Akt (Ser473), anti-Phospho-Akt (Thr308), anti-Akt, anti-Phospho-ERK1/2 (Thr202/Tyr204), anti-ERK1/2, anti-phospho-c-Raf (Ser338) were from cell signaling technology (Woburn, MA, USA).

### 4.2. Cell Culture

PC12 cells were cultured as previously described [33]. Cells were maintained and grown in DMEM culture medium containing 10% fetal bovine serum (FBS). 100 μg/L streptomycin and 100 U/mL penicillin were added into the medium to avoid contamination. Cells were kept at 37 °C in a humidified atmosphere containing 5% CO_2_. Cells were passaged when they reached about 80–90% confluency. 24 h before treatment, cells were seeded to plates which were pre-coated with poly-l-Lysine by DMEM containing 1% FBS. The SH-SY5Y cells were cultured using the same protocol as PC12 cells.

### 4.3. Cell Treatment

To study the effect of IGF-1 on the cell proliferation of PC12 cells, cells were treated with IGF-1 (1–100 μg/L) under serum-deprived conditions. Cell proliferation was measured by MTT assay or CCK-8 assay. To explore the antagonistic effects of TSN on IGF-1, PC12 cells were pretreated with TSN and then IGF-1 was added into the medium. 24 h after treatment, the cell viability was detected by MTT assay. For western blot analysis, the medium for PC12 cells or SH-SY5Y cells were replaced with DMEM 4 h before reagents were added [44]. To study the effect of IGF-1 on the phosphorylation of IGF-1R, PC12 cells were treated with 10 μg/L IGF-1 for various times (5–80 min) or various concentrations of IGF-1 for 40 min. To investigate the inhibitory effects of TSN on IGF-1R, cells were pretreated with TSN for 40 min prior to the application of IGF-1.

### 4.4. MTT Assay

Cell proliferation was evaluated by the MTT assay [25]. In brief, PC12 cells were seeded in a 96-well plate. After incubation overnight, the medium was replaced with fresh DMEM, and then cells were exposed to IGF-1 and/or TSN for 24 h. MTT (0.5 mg/mL) was added into the each well and incubated for another 4 h at 37 °C. After incubation, cells and MTT formazan crystals were solubilized by DMSO and absorbance was measured at 570 nm with a plate reader.

### 4.5. CCK-8 Assay

Cells were treated the same way as for MTT assay. In brief, PC12 cells were seeded in 96-well plates at a density of 1 × 10^5^ cells/cm^2^ and 100 μL medium for each well. 24 h after treatment with IGF-1 or IGF-1 plus TSN, 10 µL of the CCK-8 solution was added to each well followed by incubation for another 2 h. The absorbance at 450 nm was measured using a spectrophotometer (Bio-Rad, Hercules, CA, USA).

### 4.6. Flow Cytometry Assay

Flow cytometry was used to assess cellular apoptosis. PC12 cells were seeded into 6-well plates. After treatment with TSN, the cells were harvested and washed twice with ice-cold PBS and suspended with Annexin V binding buffer. The cell supernatant was stained with 5 µL Annexin-V-FITC and 15 µL propidium iodide solution. The number of apoptotic cells was analyzed using a flow cytometry. Each experiment was repeated three times.

### 4.7. Western Blotting

Western blotting was performed as described previously [33,44]. Briefly, cells were harvested and lysed in RIPA buffer containing phosphatase inhibitors and protease inhibitor cocktail at 4 °C for 1 h. After centrifugation, the supernatant was collected for determining the protein concentration. Samples were then boiled for 8 min, and equal amounts of protein (30 μg) were separated by SDS-PAGE on 10% Tris-glycine gels. The proteins were then transferred to polyvinylidene difluoride membranes. After incubation with 5% nonfat milk at room temperature for 2 h, phospho-IGF-1R, IGF-1R, phospho-ERK1/2, ERK1/2, phospho-Akt, Akt, phospho-FoxO1, phospho-FoxO3a, phospho-GSK-3β, phospho-c-Raf and GAPDH were determined by incubating with their specific primary antibodies overnight at 4 °C. After washing with TBST thrice at room temperature, the membranes were incubated with peroxidase-conjugated secondary antibody (1:5000) for 1 h. Membranes were then washed again with TBST three times and visualized using the enhanced chemiluminescence system (ECL system). The relative density of the protein bands was quantified using Image-J.

### 4.8. Statistical Analysis

Data is expressed as mean ± S.E.M. Analysis of variance (ANOVA) with Dunnett’s test was used to establish statistical significance set at *p* < 0.05. Statistical analyses were performed using SPSS 13.0 for Windows (SPSS software, SPSS, Chicago, IL, USA). 

## Abbreviations

Akt	protein kinase B
CCK-8	cell counting kit-8
CDKs	cyclin-dependent kinases
DMSO	dimethyl sulfoxide
ERK1/2	extracellular-signal related kinase 1/2
FOXO1	forkhead box O1
FOXO3a	forkhead box O3a
GSK-3β	glycogen synthase kinase-3β
IGF-1	insulin like growth factor 1
IGF-1R	insulin like growth factor 1 receptor
JNK	Jun-amino-terminal kinase
MAPK	mitogen-activated protein kinase
mTOR	mammalian target of rapamycin
MTT	methyl thiazolytetrazolium
p70S6K	ribosomal protein S6 kinase
PC12	pheochromocytoma
PI3K	phosphatidylinositol-3-kinase
TSN	Tanshinone IIA
